# Two-arm randomised futility trials: PD-stat - a futility trial of a potential neuroprotective treatment in people with Parkinson's disease

**DOI:** 10.1186/1745-6215-16-S2-P236

**Published:** 2015-11-16

**Authors:** Siobhan Creanor, Jane Vickery, Vicky Eyre, John Zajicek, Sue Ball, Jordan Elm, Camille Carroll

**Affiliations:** 1 Plymouth University, Plymouth, UK; 2 University of St Andrews, St Andrews, UK; 3 University of Exeter, Exeter, UK; 4 Medical University of South Carolina, South Carolina, USA

## 

Futility trials are efficient, early phase studies designed to eliminate potential interventions or treatments before moving into large and expensive, definitive phase III trials. In a futility trial, the null and alternative hypotheses are reversed in comparison with the usual superiority trial and are one-sided. The null hypothesis is that the intervention reaches or exceeds the required level of benefit.

Initially used in oncology, where such studies are usually simply referred to as phase II trials and have relatively short follow-up periods, there has been increasing interest in the use of the futility trial design in other clinical areas, in particular in neurological diseases. Typically, such futility studies have had a single arm and have tested whether the new treatment exceeds a pre-defined futility threshold, set as the minimum response worthwhile to justify moving to a definitive trial. However, in neurological diseases, such as Parkinson's disease, the lack of a concurrent control group has led to criticism of the subsequent findings. In an attempt to overcome such issues, it is possible to use a randomised two-arm design, whilst still testing for futility.

PD-STAT, funded by the Cure Parkinson's Trust (CPT) and JP Moulton Charitable Foundation as part of the CPT's Linked Clinical Trials Initiative, is the first UK study to utilise this design in Parkinson's disease. We will describe the design characteristics and planned analysis of this trial and outline some of the advantages and challenges associated with futility trials.

